# Willingness to accept capitation payment system under the Ghana National Health Insurance Policy: do income levels matter?

**DOI:** 10.1186/s13561-017-0175-1

**Published:** 2017-11-03

**Authors:** Frank Gyimah Sackey, Peter N. Amponsah

**Affiliations:** Catholic University College of Ghana, Sunyani, Ghana

**Keywords:** Capitation payment, Health insurance, Diagnosis-related grouping, Ghana

## Abstract

This research was set to examine the factors influencing the willingness and the likelihood of Ghanaians to accept the capitation payment system under the National Health Insurance Scheme. Data was collected through the random sampling method in all the ten regions of Ghana. A probit estimation with marginal effects was adopted to examine the factors influencing the willingness and the likelihood while the generalized Blinder-Oaxaca decomposition was used to examine the extent to which individual characteristics influence the acceptance gap between high income and low-income earners. Our results indicated that, at the individual level, high income, being employed, awareness and smaller household size were the significant factors influencing the willingness and the likelihood to accept capitation. We also observed that the acceptance gap between high income and low-income earners was largely influenced by unexplained factors other than individual characteristics of high income earners. Since the willingness and the likelihood to accept capitation are mixed across regions and largely dependent on high incomes, the intention to roll out and implement the capitation system should be as a complementary payment system to the already existing one, being the diagnosis related grouping. We recommend that the current payment system, the diagnosis-related-grouping be maintained and complemented with capitation while measures to reduce, if not eliminate, the abuse associated with it be put in place.

**Electronic supplementary material:**

The online version of this article (10.1186/s13561-017-0175-1) contains supplementary material, which is available to authorized users.

## Background

The wealth of every nation is partly dependent on the health of its people. It is estimated that for every 10% increase in life expectancy at birth there is a corresponding rise in economic growth of 0.4%, and this economic growth leads to a further rise in life expectancy at birth [22]. According to Deaton [9], increase in growth leads to rise in incomes, which contributes to good health. Gupta and Mitra [14] observe that growth tends to reduce poverty and improves health status. They also find that growth and health status are positively correlated and have a two-way relationship. This finding suggests that better health enhances economic growth by improving productivity, and higher growth allows better human capital formation. For this reason, various governments try to put in place policies that will ensure improved health status of their citizenry. A major means of achieving this goal is to make health facilities available, accessible, and affordable. Therefore, health insurance policies have become a very important phenomenon.

Health insurance is an insurance product that insulates a person from financial loss due to accident, ill health and/or disability. Though these unexpected circumstances are not fixed and precise, their significance to society is that the condition of ill health arising from disease or injury prevents one from pursuing one’s normal routine of living and affects one’s survival. Therefore, health insurance has become a necessity; but it is not to everyone, especially the low-level income earners and poor homes in developing countries such as Ghana. Meeting the sustainable development goals (SDGs) involves making healthcare delivery accessible and affordable without compromising quality. Thus, in a bid to improving the health needs and accessibility, the government of Ghana introduced the Ghana National Health Insurance Scheme (NHIS) to provide financial access to healthcare services of which the National Health Insurance Act, 2003 was passed by parliament. The scheme has moved from paying its accredited providers through the Fee-For-Service (FFS) to the Diagnosis- Related -Grouping (DRG) as a way of dealing with the abuse of the FFS system [10]. The DRG system, however, has also some weaknesses. For example, the DRG has been cited for not being able to successfully contain cost, especially for outpatient claims. In fact, outpatient claims account for 70% of the total NHIS claims and 30% total cost, which lead to a huge cost burden on the Authority (HIP, 2010). Such problems have led to the overhaul of its provider payment system with the introduction of the capitation payment which is currently being introduced as a pilot program in some selected regions across the country.

The NHIS law that established the National Health Insurance in Ghana provided for the institution of multiple payment methods including capitation. LI 1809, specifically mentions capitation as one of the provider payment methods to be considered for use under the NHIS. This has been the international best practices given the fact that no single provider method has been perfect. The proposed reform in Ghana does not do away with any of the already existing provider payment methods. Rather it introduces capitation for a specific level of care – the primary level of walk in outpatient care, which is the fundamental base of the health care systems, and reserves the DRG for services and Itemized Fee for medicines system to the higher levels of care. Under the proposed capitation system, the amount paid to providers will cater for selected OPD primary care cases.

The advantages of introducing per capita payments for first level outpatient primary care, as a complementary payment method to the already existing methods in the Ghana NHIS, include the following: It will reduce the current massive administrative and staff time costs of claims preparation, submission, vetting and reimbursement involved in using G-DRG and fee for services for medicines to pay for first line OPD care, It will improve the ability of the NHIA to forecast and budget, It will eliminate problems of delayed payment of claims – for the services in the per capita basket. This is because monies are now being paid in advance to providers. By tying clients to a PPP of their choice it reduces fragmentation of care and introduces continuity of care for clients. It will also enable proper implementation of a referral system. By enforcing the implementation of the gatekeeper system – which is already part of the policy of the Ministry of Health, it will reduce some of the current misuse of care and resultant costs and wastage. For example under the current system a client can visit several providers with the same condition – even on the same day, consuming staff resources and medicines at each point. This is a duplication and waste of scarce staff and financial resources. The sharing of risk between schemes/NHIA, providers and clients under a per capita system has a better potential to ensure the financial sustainability and preservation of the NHIS.

The major disadvantage of a per capita system is that the provider may be tempted to provide less than needed services to the client. A close monitoring of quality of care is therefore essential in a per capita payment system. It is also necessary to continuously monitor the per capita rate to make sure that it is and remains fair for the package of services covered. An examination of the costs to providers and the NHIS in terms of skill and staff time in claims processing suggested the need to revisit and work out a way of implementing the capitation payment method, which was proposed for use in Ghana at the time the LI 1809 that accompanied Act 650 was drawn but which remained unimplemented.

In summary the payment-method reforms involve: Introducing capitation (per capita payment) to replace DRG for service & FFS for medicines at the primary care level, retaining DRG for Services & Fee for service (FFS) for medicines at Specialist OPD clinics, Hospital referrals and inpatients (district, regional, specialist and teaching hospitals). The reforms are supported by: Accountability (financial management and reporting systems), strengthening of routine management information systems data completeness, quality, analysis and use, built in monitoring and evaluation for continuous quality improvement, improving clarity in stakeholder roles and relationships, and communication.

Ghana’s objective in introducing capitation into its provider payment systems under the NHIS stem out of the already described advantages of capitation which are to: improve cost containment and viability of NHIS, share financial risk between schemes, providers and subscribers, introduce managed competition for providers and choice for patients (compatible with portability) to increase the responsiveness of the health system, improve efficiency and effectiveness of health services through more rational resource use, correct some imbalances created by the G-DRG, e.g., OPD supplier-induced demand, simplify claims processing, address difficulties in forecasting and budgeting, better provider-patient relationship.

The capitation system, which began in the Ashanti Region in 2012 on a pilot basis, has since been extended to the Volta, Upper East, Upper West, Brong Ahafo, Central, and the Western Regions (NHIA, 2015). The system has six main technical components: the package of services paid through the per capita rate, the base per capita, adjustment coefficients, enrolment/registration, financial management and reporting system, and quality monitoring system. These technical components would purportedly check and deal with all the problems associated with the payment system.

Besides eliminating the inefficiencies in the capitation payment system, the system has received protests from civil society organizations, politicians and service providers for its perceived negative effects on primary care delivery (Owusu-Sekyere and Bagah, 2014). In a press release, the Society of Private Medical and Dental Practitioners notified the public that it has suspended its services to the NHIS subscribers indefinitely. According to the medical and dental practitioners, the system is detrimental to quality of health care provision and a major threat to the survival of private health facilities [19]. Moreover, the Pharmaceutical Society of Ghana is reported to have sued the NHIA over capitation payment on grounds that it poses grave danger to patients given, among other factors, the recognition it (capitation payment) gives to persons outside the pharmacy profession (The Daily Guide, 2012). Hence, the capitation payment continues to be a subject of public discussion in Ghana.

According to a USAID report [21], some of the reasons for the rejection of the capitation system by the Ashanti region was the exclusion of primary care maternity services, i.e., antenatal care, normal delivery and post-natal care, the wide variation in primary care service quality and availability were not addressed. While proponents rationalize its importance and usefulness for health care delivery in Ghana, opponents call for its cessation [3]. The National Health Insurance Authority is currently sensitizing major stakeholders in the Brong Ahafo Region to enable them to understand the operation of the capitation system so that they assist in the dissemination of information to the people. Such sensitization and education may contribute to stakeholders’ acceptance of the policy and its effectiveness in increasing accessibility, quality and sustainable healthcare delivery.

### Statement of the problem

The capitation payment system under the NHIS has received opposition especially from providers and civil organizations. According to these groups the capitation system would adversely affect patient health outcomes because the financial risk transferred to providers may lead to under-provision of services and thereby reducing quality (Dodoo, 2013). This argument, however, does not provide any empirical evidence to support the claims. According to Agyei-Baffour, Oppong and Boateng [1], anytime there is an introduction of any new system or change, the fear of the unknown and protection of interests create anxiety among parties, and this apprehension may lead to misconception on the consequences of such new system. Also, according to Gibson et al. (2003), the resistance mainly arises from the limited attention paid to communicating the change to actors. However, such countries as the UK, Thailand and Chile have successfully implemented the capitation system of payment by increasing access and improving quality service delivery Allard et al. [2]. Therefore, it is important for policy makers to manage the interest of actors to arrive at desired policy goals and objectives.

Since 2012 when the pilot program for the capitation began in the Ashanti region, there have been various sensitization programs aimed at creating awareness. At the same time, resistance from providers has persisted. Whereas the resistance to the system comes from the providers, the opinions of the main people whom the policy seeks to protect and ensure that they received quality healthcare service have not been well engaged to determine their willingness to accept this system of payment. So, it is timely and relevant for this study to determine the public’s willingness to accept the capitation as a system of payment under the National Health Insurance Scheme and the factors influencing this willingness or otherwise. In this regard, this study conjectures that the willingness of the people to accept capitation will depend on the citizens’ individual and socio-economic characteristics, as well as their ability to afford and subscribe to this system of payment.

### Objectives

Objectives for this paper include:

1. To determine the factors that influence the willingness to accept the capitation payment system under the Ghana National Health Insurance Scheme.
2. To examine the extent to which income levels influence the willingness to accept the capitation payment system under the Ghana National Health Insurance Scheme.

This study’s outcomes and recommendations may inform policy makers about the various factors influencing people’s willingness to accept and subscribe to the capitation payment system. This paper will also contribute immensely to the body of literature under health insurance for health practitioners, service providers, researchers, and policy makers generally.

The first part of this paper provides an overview of health insurance policy and states the problem regarding the capitation system of payment, the research objectives and significance of the study. The second section deals with the conceptual framework and literature on health insurance provision and schemes in Ghana. The next section discusses the method of collecting the data for the study and analyzing the data. The study proceeds with the econometric analysis of the data, as well as the presentation and discussion of results. Lastly, the study draws some conclusions and makes recommendations.

### Review of literature

#### Health insurance

Health insurance is a way of pre-paying for the health services used by residents. In health insurance, payments made are spread over the subscribers and over time in the form of some agreed regular contribution. For an efficient health insurance system, the following issues need to be addressed; how money is collected from residents and pooled to pay for services, the services to be covered under the insurance or the benefit package and how these services are paid for on behalf of the citizens who are part of the insurance scheme (also known as the provider payment method. The provider payment method is therefore “the mechanism used for transferring funds from the purchaser of healthcare services to the providers”.

Different methods are used to pay providers under a health insurance scheme. They include the Fee for Services (often itemized), the Diagnosis Related Groupings (DRG) and Capitation. There is no one method that is perfect as each method has its own advantages and disadvantages. Therefore a skillful mix of methods, taking into consideration the unique characteristics of the country, is required.

#### The fee for service

Under the Fee for Service provider payment method, the provider lists the different services which they have provided for the client and the cost of each service and requests payment. The advantage of the fee for service payment method is that the provider has no incentive to leave anything off the “shopping list”. The disadvantage is that precisely for this reason, since the provider is also often the “owner” of the shop and the one choosing the items to be purchased for the client (because of the issues related to specialized knowledge); it is possible for the provider to provide unnecessary services to maximize profit. It does not mean that the provider will do this, but it is possible and hence, a known weakness of this system.

Experience all over the world over time shows that it is a system that can lead to cost inflation of costs and threaten the sustainability of health insurance. Countries that use Fee for Service successfully in their health insurance scheme, an example of which is Germany, often devise very complicated methods to counteract this tendency. Despite its well established disadvantage of causing cost inflation which can be a major threat to sustainability - and sometimes leading to unnecessary provision of services because it requires very little technical expertise to implement - fee for service is a common payment mechanism in many low income countries (Ghana National Health Insurance Authority).

#### Diagnosis related grouping (DGR)

With regards to the DRG payment method, related diagnoses are grouped together and the average cost of treatment in that group determined. Providers are therefore paid according to the diagnosis they give their client. Many developed countries including the USA and U.K, use DRG as part of their payment systems. Providers have to fill claims forms for reimbursement after providing the services. The claims made by the providers are then checked (vetted) for accuracy and genuineness before payment. The process is administratively complicated and makes a heavy demand on the time of both provider and scheme staff and the NHIA. The DRG for services also still holds some incentives for cost escalation though they are less, compared to the itemized fee for service. Since medicines at all levels remain under itemized fee for service, the potential of major cost escalation is also strong.

#### Capitation

Capitation is a provider payment mechanism in which providers in the payment system are paid, typically in advance, a pre-determined fixed rate to provide a defined set of services for each individual enrolled with the provider for a fixed period of time. The amount paid to the provider is irrespective of whether that person would seek care or not during the designated period.

The fixed amount is typically expressed on a Per Member Per Month (PMPM) basis. The member refers to NHIS subscribers assigned to the accredited providers. Under this payment system, the member or subscriber selects a preferred primary provider (PPP) to provide all the services under the capitation basket in exchange for the capitation rate. The total capitation amount is transferred to the provider at the beginning of the service period. The amount is calculated based on the total number of members who have selected a given provider (Ghana National Health Insurance Authority).

Capitation is a well-established provider payment method in several countries – high as well as middle income. In introducing capitation, Ghana is walking a tried and tested road that many other countries have already successfully walked. The British National Health Service has used capitation for decades. The British system has become more complicated over time with several generations of reform but the basic principle is one of capitation. Thailand, which is lauded internationally as a middle income country that now successfully covers virtually all its citizens with health insurance, uses capitation as the base of its provider payment system and reserves methods such as DRG for the higher referral level. Other examples of middle income countries successfully using capitation as one of their provider payment methods include Chile and Estonia, and have been successful in attaining universal or near universal coverage with health insurance.

### Institutional framework

#### The Ghana health service

The Ghana Health Service (GHS) is a public service body established under Act *525* of 1996 as required by the 1992 Constitution. The GHS does not include teaching hospitals and private hospitals. It is an autonomous executive agency responsible for implementation of national policies under the control of the Minister for Health through its governing council – the Ghana Health Service Council. However, the GHS continues to receive public funds and thus remains within the public sector. Yet, its employees are no longer part of the civil service. Neither are the GHS managers required to follow all civil service rules and procedures. This independence of the GHS is designed primarily to ensure that its employees have a greater degree of managerial flexibility to carry out their responsibilities than would be possible if they had remained wholly within the civil service. The mandate of GHS is to provide and prudently manage comprehensive and accessible health service with special emphasis on primary health care at the regional, district and sub-district levels in accordance with approved national policies [12].

The objectives of the GHS involve: implementing approved national policies for health delivery in the country, increasing access to good quality health services, and managing prudently resources available for the provision of the health services. For the purposes of achieving these objectives, the GHS performs the following functions amongst others: provide comprehensive health services at all levels directly and by contracting out to other agencies, which includes developing appropriate strategies and setting technical guidelines to achieve national policy goals; undertake management and administration of the overall health resources within the service; promote healthy mode of living and good health habits by people; establish effective mechanism for disease surveillance, prevention and control; determine charges for health services with the approval of the Minister of Health; provide in-service training and continuing education; perform any other functions relevant to the promotion, protection and restoration of health [12].

Ghana introduced its National Health Insurance Scheme (NHIS) in 2003 through an Act of Parliament (Act 650) as a result of the inability of majority of Ghanaians, mostly the poor, to access health care under the Cash and Carry System of health care financing. It is said that only 21% out of 18% of Ghanaians who needed health care under the Cash and Carry System could afford to pay at the point of services. This estimate means that about 79% of people who needed healthcare could not afford (Send [18]).

In order to promote universal coverage and equity, the government of Ghana adopted the National Health Insurance Scheme (NHIS) in 2003, which was fully implemented in 2005. Under the law, there is a National Health Insurance Authority which licenses, monitors and regulates the operation of health insurance schemes in Ghana. The NHIS aims at ensuring equitable and universal access for all residents of Ghana to an acceptable quality package of essential health care services without out-of-pocket payment being required at the point of use [13]. Thus, the ultimate goal of the NHIS is the provision of universal health insurance coverage for all Ghanaians, irrespective of their socio-economic background. As at June 2009, about 67% of the Ghanaian population had subscribed to the NHIS [5].

The NHIS is based on District Mutual Health Insurance Scheme (DMHIS), which operate in all 170 districts of the country. The NHIS covers both the formal and informal sectors of the economy. According to McIntyre, Gilson and Mutyambizi [15], the implementation of the NHIS draws experience from the operations of the Community Based Health Insurance Scheme (CBHIS). The NHIS is financed by a national health insurance levy of 2.5% on certain goods and services, 2.5% monthly payroll deduction being part of the contribution to the Social Security and National Insurance Trust (SSNIT) for formal sector workers, government budgetary allocation, and donor funding. But the formal sector workers still have to pay a registration fee to a DMHIS for an identity card (ID) to be able to access health care services. Also, contributions from members of the informal sector are made to the NHIS with the minimum and maximum premium being GH₵ 7.20 ($1.75) and GH₵ 47.70 ($11.93), respectively [13]. However, the core poor, pregnant women, pensioners, people above the age of 70 and those below 18 years are exempted from premium payment. There is no other cost sharing or co-payments with the NHIS, except the premium paid.

The benefits’ package of the NHIS consists of basic health care services, including outpatient consultations, essential drugs, inpatient care and shared accommodation, maternity care (normal and caesarean delivery), eye care, dental care, and emergency care. About 95% of the diseases in Ghana are covered under the NHIS. However, some services classified to be very expensive are on the exclusion list. Among these are cosmetic surgery, drugs not listed on the NHIS drugs list (including antiretroviral drugs), assisted reproduction, organ transplantation, and private inpatient accommodation [8].

#### The provider payment method

There is no one perfect method; each method has advantages and disadvantages. Typically, therefore, most successful health insurance schemes use a combination of methods. A skillful mix of methods considering each country’s unique context, including economics and history, is the best approach. Thus, effectively and efficiently managed health insurance schemes often provide for a mix of provider payment methods in a way that allows the advantages and disadvantages of the different methods to balance each other (NHIS, 2011). Capitation payment methods are able to control costs better than DRGs, but capitation can encourage providers to deliver less health care than optimal. On the other hand, DRG payments focus on technical efficiency to make better use of available resources and reduce average length of stay, but they also encourage hospitals to increase patient numbers [20].

In Ghana, provider payment methods in use currently are (a) Itemized Fee for Service (FFS) for noninsured clients for both services and medicines, (b) Diagnosis Related Groupings (DRG) for insured clients (services only), and (c) Itemized Fee for Service (FFS) for medicines of insured clients. However, these methods of payment have suffered abuses by service providers as well as subscribers. It has been observed that subscribers often patronize several health facilities with the same ailment within a period less than a month. Often, claims submitted by providers would have an individual with the same NHIS registration number appearing on more than one service provider’s claim with the same ailment and similar drugs. Reasons like these have motivated the government of Ghana to implement the capitation payment system.

In 2010, the World Bank supported Ghana to introduce the capitation payment system as part of the National Health Insurance services. According to Ansong [4], the National Health Insurance Scheme in Ghana selected Ashanti region for the pilot of the capitation payment system because the region is situated in the middle of the country and has heterogeneous infrastructure and culture.

### Empirical literature

Andoh-Adjei et al. [3] observed that health insurance subscribers in Ghana have high trust in their primary care provider for giving them quality care under capitation payment despite their negative attitude towards capitation payment. The study also added that subscribers are guided by proximity and quality of care considerations in their choice of provider. In contrast, Benoah [6] assessed the effects of the capitation payment system on health care delivery in Ashanti Region and found that the majority of subscribers (representing 90% of the respondents) admitted that they were not satisfied with the healthcare delivery under capitation. Hence, they did not see any improvement and effectiveness in healthcare delivery in the Ashanti region. This finding, however, is also different from the findings of Opoku, Nsiah, Oppong and Tetteh [17] who undertook a similar study within the same Ashanti region and observed that the capitation payment system has an efficient use of resource, creates competition among hospitals (quality service), and has turned hospitals to financial risk bearers.

However, an earlier study by Agyei-Baffour, Oppong and Boateng [1] on knowledge, perceptions and expectations of capitation payment system in a health insurance setting indicates that a majority (97.9%) of the clients had heard of the capitation payment. But, that knowledge did not reflect the clients’ level of understanding of the system. About two-thirds (61.2%) disclosed that capitation was not important for them as clients were restricted to one Preferred Primary Provider (PPP) for a long period of time. Health providers also believed that people (34%) did not like the capitation payment system due to their misconception that it has been politicized. Others, constituting 26%, believed that the system did not give clients free choice of providers, and 17% held that capitation did not cover most drugs. Although awareness of the capitation was high among clients, attitudes toward the capitation payment system were somewhat poor.

The studies suggest that a good understanding of the capitation payment system is key to ensuring client and provider acceptance, as well as a smooth implementation of the system. It must be emphasized that almost all the studies about capitation payment system in Ghana have been on perception and expectations of the subscribers. None determined the extent to which subscribers’ socio-economic and individual characteristics influenced their willingness to accept the capitation as a payment system under the Ghana Health Insurance Scheme. Thus, this study shifts the focus to examining the willingness of subscribers to accept the capitation payment system based on clients’ individual and socio-economic characteristics.

## Methods

### Data

A sample of 1299 NHIS subscribers who have reached the working age and are either employed or unemployed from all the ten regions of Ghana were used for the study. The number of questionnaires administered for each region was chosen proportionately based on regional population as given by the 2010 Ghana Statistical Service Population Census [11]. The questionnaires were then administered randomly with the regional distribution as indicated in Table 1.

**Table 1:** Regional population and sample distribution

Region	Population	Sample size	Percentage
Ashanti	4,780,380	252	19.4
Greater Accra	4,010,054	211	16.24
Eastern	2,633,154	139	10.7
Northern	2,479,461	130	10.01
Western	2,376,021	125	9.62
Brong Ahafo	2,310,983	122	9.4
Volta	2,118,252	112	8.62
Central	2,201,863	116	8.93
Upper East	1,046,545	55	4.23
Upper West	702,110	37	2.85
Total	24,658,823	1299	100

Households were chosen at random, and questionnaires were administered to household heads. In the questionnaires, household heads were asked to answer questions with regard to their individual characteristics, their income levels, employment status, whether they have received education or sensitization about the capitation payment system and hence their perception and willingness to subscribe to the capitation payment system. Household heads were chosen for the study because they will, to a large extent, be responsible for the payment of their defendants under the payment methods.

To effectively examine the success of the capitation payment system, service providers and physicians were interviewed to solicit their views on the payment system. Thirty-three (33) were interviewed with at least three (3) providers from each region.

### Descriptive analysis of data

Table 2 shows the descriptive analysis of the data. Out of the 1299 respondents used for the study, 571 (43.96%) were female while 728 (56.04%) were male. A total number of 765 (58.89%) were employed whereas 534 (41.11%) were unemployed or perceived themselves as unemployed though they were doing menial jobs for a living. In terms of their educational background, 689 (53.04%) of respondents had attained tertiary level of education, 255 (19.63%) had received secondary education, 328 (25.25%) had primary education but 27 (2.08%) had no formal education.

**Table 2:** Descriptive analysis of data

Variable	Description		Mean	Std. dev	Max	Min
Income	Average monthly income of respondent		1347.2	1404	200	8000
Household size	Number of persons living in the home of respondent		5.34	4.84	1	13
Education of respondent	Education	Number/percentage				
	Tertiary	680(53.04)				
	Secondary	255(19.63)				
	Primary	328(25.25)				
	Illiterate	27(2.08)				
SEX	Gender	Number/percentage				
	Female	571(43.96)				
	Male	728(56.04)				
Age	Age of respondent		38.44	8.32	20	63
Location	Location	Number/percentage				
	Rural	851(65.51)				
	Urban	448(34.49)				
Awareness	Knowledge	Number/percentage				
	No knowledge	956(73.60)				
	Aware	343(26.40)				
Employed	Employment	Number/percentage				
	unemployed	534(41.11)				
	employed	765(58.89)				
Marital status	Marital status	Number/percentage				
	Single	516(39.72)				
	Widow	65(5)				
	Divorced	93(7.16)				
	Married	625(48.12)				

On whether respondents have any knowledge or awareness of the government’s intention of implementing the capitation payment system under the Ghana National Health Insurance Scheme, 956 (73.60%) indicated that they have knowledge or awareness of the capitation policy while 343 (26.40%) did not. The mean age of the respondents was 38.44; the youngest was 20 years of age, and the oldest was 63 years. A total number of 448 (34.49%) lived in rural areas, categorized as towns with only Community-Based Health Planning and Services (CHPS) compound health facility, but 851 (65.51%) lived in the urban centers. With regard to their marital status, 516 (39.72%) were single whereas 65 (5%) were widows and 93 (7.16) were divorced. However, 625 (48.12%) were married.

### The empirical model

#### The willingness to accept the capitation as a system of payment

The willingness to accept the capitation payment system of the NHIS is estimated by a linear model as follows:

1 $$ {W}_i={\beta}^{\hbox{'}}{X}_i+{\varepsilon}_i $$

Where *W*_*i*_ is the willingness which is the outcome variable denoted by “1” if an individual *i* is willing to accept the capitation payment system and “0” if otherwise. The matrix *X* contains a set of variables picking up the respondent’s individual, social and economic characteristics.

These are the explanatory variables consisting of age(respondent’s age), income (the respondent’s income level), sex (the gender of the respondent, denoted as “1” if male and “0” if female), household size (respondent’s household size), education(level of education attained by respondent ranked as “4” if tertiary, “3” if secondary, “2” if primary and “1” if illiterate), marital status(marital status of the respondent ranked as “4” if married, “3” if divorced, “2” if widow and “1” if single), awareness(whether respondent knows about capitation denoted as “1” if yes and “0” if otherwise), preference (if respondent prefer capitation to other form of payment denoted “1” if yes and “0” if otherwise), employed (employment status of the respondent denoted as “1” if employed and “0” if unemployed), location (area of residence of respondent denoted as “1” if urban and “0” if rural), region (a dummy for each of the ten regions). Eq. (1) shall be estimated using the linear probability model (LPM) with the stata version 12 software.

To determine the extent to which income level influences the willingness to accept capitation, income shall be differentiated by high and low-income earners. Here high-income earners shall be defined as individuals whose incomes are greater or equal to the average income based on the data while low incomes are those below the average. Therefore, the study seeks whether it is the individual characteristics of high income earners that influence their willingness to accept capitation or just their income levels. To do this we resort to the Blinder-Oaxaca decomposition  [7, 16].

The empirical model to be estimated is of the form:

2 $$ Willingness=\alpha +{\beta}_1 Ylevel+{\beta}_2 hsize+{\beta}_3 edu+{\beta}_4 sex+{\beta}_5 age+{\beta}_6 loc+{\beta}_7 aware+{\beta}_8 empl+{\beta}_9 pref+{\beta}_{10} region+{\varepsilon}_i $$

Where *Ylevel*, is the income level, *hsize*, the household size, *edu*, the educational level attained, *sex*, the gender of respondent, *age*, the age of respondent, *loc*, residential area of respondent, *aware*, whether the respondent knows about the capitation, *empl*, employment status of respondent, *pref*, whether respondent prefers capitation to other forms of payment, *region*, region of residence of respondent and *ε*, being the error term. Ashanti region was used as the base category for the following reasons: the region is found in the middle belt of Ghana and has been a major destination center for people migrating from both the northern and southern part of the country. The region also has the largest population size. It was the region where the pilot program was first rolled out, and the fact those civil society organizations and some service providers in the region were in resistance to its implementation.

#### Determining the extent to which income levels influence the willingness to accept capitation

To determine the extent to which income levels influence the willingness to accept capitation, we rewrite Eq. (1) as follows:

3 $$ {W}_{ij}={\beta}_{ij}+{\varepsilon}_{ij} $$

In Eq. (3) the subscript ij reflects the belongingness to a particular group, e.g., high-income earners and low/non-income earners. Assuming that “*h*” is for high income earners and “*I*” is for low income earners with marginal effects *β*_*h*_ and *β*_*l*_ respectively, differences in the willingness to accept capitation will exist if *β*_*h*_ ≠ *β*_*l*_.

The necessary assumption being made is that the error *ε*_*ij*_ is independent of individual *i* belonging to group *j* = *h* or *j* = *l*. Assuming *β*_*h*_ and *β*_*l*_ are the coefficient for employment for example then differences in acceptance will require that |*β*_*h*_| > |*β*_*l*_|or vice versa. The difference would be attributed to the fact that one group, e.g., high income earners, is endowed with a set of variables (characteristics) that makes high income earners likely to accept capitation than low income earners or vice versa. Thus *X*_*h*_ ≠ *X*_*l*_.

To properly quantify the extent to which the variables *X*_*ij*_ (endowments) and coefficient *β*_*ij*_ (unexplained factors) influence the decisions to accept capitation we resort to the Blinder-Oaxaca decomposition method. This method allows us to decompose the estimated acceptance gap between high income (*A*_*h*_) and low/non-income earners (*A*_*l*_) into two components as follows:

4 $$ {\widehat{A}}_h-{\widehat{A}}_l=\left({\overline{X}}_h-{\overline{X}}_l\right){\widehat{\beta}}_l-{\overline{X}}_h\left({\widehat{\beta}}_h-{\widehat{\beta}}_l\right) $$

The left hand of Eq. (4) measures the acceptance gap (estimate gap). The first term on the right-hand side of the equation picks up that part of the gap attributed to differences in endowments(characteristics), whilst the second term picks up the part attributed to coefficients (unexplained factors). Where the first part on the right-hand side is greater than the second part (in absolute terms), the decision to accept capitation by high income earners is largely influenced by their characteristics. On the other hand, where the second term on the right-hand side is greater than the first term (in absolute terms) then this acceptance gap is being largely influenced by unexplained factors. That means that high income earners’ acceptance of that capitation is not influenced by their characteristics such as age, gender, employment status, marital status, etc., but just the fact that their incomes are high.

## Results and discussion

### Willingness to accept capitation

Table 3 shows the estimated effects of the likelihood that an individual and a region will accept the capitation payment system under the Ghana National Health Insurance Scheme. To allow for interpretation the study reports the marginal effects instead of coefficients.

**Table 3:** Willingness to accept capitation: probit estimation with marginal effects

Willingness	dF/dx	Std. Err	z	P > |z|	x-bar	[95% C.I.]
Log (Income)	.0850317	.015751	5.38	0.000	7.65901	.05416 .115903
Second Cycle	.0465709	.0433296	1.09	0.275	.196305	−.038354 .131495
Primary School	.0034488	.0380682	0.09	0.928	.252502	−.071164 .078061
Illiterate	.1024493	.1116649	0.95	0.340	.020785	−.11641 .321309
Sex	−.0170879	.0453663	−0.38	0.707	.397998	−.106004 .071828
Age	.004945	.0027895	1.77	0.076	38.4434	−.000522 .010412
Awareness	.2526399	.03664	6.98	0.000	.264049	.180827 .324453
Employed	.0954805	.0282816	3.39	0.001	.411085	.04005 .150911
Preference	.0113417	.0319956	0.35	0.723	.482679	−.051369 .074052
Widow	−.0178344	.0660746	−0.27	0.790	.050038	−.147338 .111669
Divorced	−.0587199	.0390965	−1.47	0.140	.30254	−.135348 .017908
Married	.0346609	.0453723	0.77	0.440	.250192	−.054267 .123589
Location	.0500246	.0300452	1.68	0.093	.344881	−.008863 .108912
Household size	−.0106649	.0036245	−2.94	0.003	5.34411	−.017769–.003561
Greater Accra	.2292637	.0518286	4.52	0.000	.163972	.020675 .271579
Eastern Region	.0198648	.0606954	0.33	0.741	.107005	−.177789 .068098
Northern Region	−.064766	.0558291	−1.11	0.267	.101617	−.245349–.016541
Western Region	.1459874	.0692565	2.19	0.029	.076212	−.08569 .212323
Brong Ahafo	.073684	.0591484	1.28	0.200	.094688	−.130942 .120848
Volta Region	.0791279	.0644581	1.26	0.206	.193995	−.183618 .036169
Central Region	.2694488	.0640492	4.25	0.000	.089299	.03834 .332441
Upper East	.4209035	.0707998	5.27	0.000	.04234	.177327 .513209
Upper West	.2635957	.0814817	3.27	0.001	.04388	.000961 .357182

At the individual level, the results show that increase in income increases the willingness and the likelihood of accepting the capitation by 8.5 percentage points. Awareness has become a very vital issue if any policy is to succeed as the targeted people need to be educated and informed about the policy and its implementation. In this study, where individuals are aware about the capitation and have knowledge of it, their willingness and the likelihood to accept the capitation increase by 25 percentage points. Compared to their unemployed counterparts, the employed are likely to accept the capitation while people from large households are less likely to accept the capitation system of payment. Of course, this is not surprising because the capitation is likely to drain incomes of bread winners of large households.

At the 10% level, people living in urban centers are more willing and likely to accept the capitation compared to their counterparts in the rural settings. This finding supports that of Andoh-Adjei et al. [3] that subscribers are guided by proximity and quality healthcare considerations. These attributes are found in the urban centers, where cluster of hospitals provide quality care and residents have ease of proximity.

At the regional level, and using the Ashanti region as the base category, the results indicated that people in the Greater Accra, Western, Central, Upper East and Upper West regions are more willing and likely to accept the capitation while the study did not find any significance in relation to the willingness and the likelihood to accept the capitation in the Eastern, Northern, Brong-Ahafo and Volta regions. Similarly, marital status, educational background, and gender as individual characteristics were not significant in determining the willingness and the likelihood to accept capitation.

Because the Ashanti region was used as the base category, the interpretation is that the people in the Ashanti region are not willing and are less likely to accept the capitation payment system. This finding supports those of Benoah (2013) and Agyei-Baffour, Oppong and Boateng [1] as well as that of USAID [21]. These findings for the various regions make the willingness and the likelihood to accept the capitation payment system mixed at the national level and as a national policy. With regard to the interviews conducted on the service providers, it was observed that eighteen (18) service providers, making up of thirteen private (13) service providers and five (5) public service providers, were against the implementation of the capitation system because they believe that their operations would be at risk because the per capita threshold payment amount would be the same irrespective of the resources used to treat patients. Thus, they are likely to spend more than the fixed per capita amount threshold given to them by the National Health Insurance Authority.

The remaining fifteen (15) service providers, constituting six (6) private service providers and nine (9) public service providers, were in favor of the payment system as they claimed that it would help increase health efficiency and deal with bloated bills sent to the National Health Authority for reimbursement. The outcome of the views of service providers is also mixed. While some providers welcome the capitation payment policy, others are also against its implementation.

### Decomposing willingness among high and low-income earners

To examine the extent to which income levels influence the willingness and the likelihood to accept the capitation, this study resorted to a decomposition analysis. As to whether the individual characteristics of high income earners influence the willingness and the likelihood to accept capitation or otherwise, the study employed the generalized Blinder-Oaxaca decomposition and counterfactual analysis [7, 16].

Table 4 shows the overall results of the generalized Blinder-Oaxaca decomposition results. The results revealed a mean acceptance of 27% for low income earners and 50% for high income earners, leading to an acceptance gap of 23% in favor of high income earners. Dividing the explained (endowments) of 0.04 by the difference (gap) of 0.23, endowments (characteristics) accounted for only 18% of the acceptance gap. This 18% was the mean decrease in the acceptance gap if low income earners possessed the individual characteristics of high income earners. The unexplained (coefficients) of 0.19 showed that the unexplained factors amounted to 82% (0.19÷0.23) of the acceptance gap. Thus, applying the unexplained (coefficients) of high income earners to the low-income earners’ characteristics, the acceptance gap will decrease by 82%. The results imply that the acceptance gap between the low income and high-income earners is not influenced by the individual characteristics of high income earners but largely by the fact that they earn high income irrespective of their age, gender, education or marital status.

**Table 4:** Income levels and willingness: blinder-oaxaca decomposition and counterfactual analysis

Willingness	Coef.	Robust Std. Err.	z	P > |z|	[95% Conf. Interval]
Overall					
Low Income	.2701525	.0146612	18.43	0.000	.2414171 .2988879
High Income	.5013123	.0256303	19.56	0.000	.4510779 .5515468
Difference	−.2311598	.0295273	−7.83	0.000	−.2890323 -.1732874
Explained	−.0400858	.0140411	−2.85	0.004	−.0676059 -.0125657
Unexplained	−.191074	.0315304	−6.06	0.000	−.2528724 -.1292756

## Conclusion and policy recommendations

The health of citizens contributes immensely to the wealth of every nation. Therefore, governments seek social policies that make health facilities available, accessible and affordable to the people. Health Insurance policy is one of such social policy interventions being implemented by Ghana as a way of making healthcare affordable. However, the Ghana Health Insurance Scheme has its challenges. A pilot of the capitation payment system in the Ashanti Region was a way of examining its effectiveness in dealing with abuses associated with the payment of claims under the FFS and the DRG. Although the pilot program has been extended to other regions, the capitation system has been abandoned recently in the Ashanti region.

For every policy to be effective its end users or the intended target groups need to be informed, sensitized and enlightened for them to understand, appreciate and embrace the policy wholly. It is also important to study their socio-economic background and individual characteristics and how these are likely to influence their willingness and likelihood of accepting such a policy. It is for these reasons that the present study sought to determine how the socio-economic as well as individual characteristics influence subscribers’ willingness and likelihood of accepting the capitation payment system as a policy. The study also included a dummy for the regions to ascertain the regions’ willingness. Another objective was to determine the extent to which income levels influence the willingness to accept the capitation system under the Ghana Health Insurance Scheme.

Using a linear probability model estimation with marginal effects, the study shows that high incomes, being employed, having smaller household size and awareness are the significant factors that increase the willingness and the likelihood of subscribers to accept the capitation payment system. At the regional level and using the Ashanti Region as a base category, the study reveals that people in the Greater Accra, Western, Central, Upper East and Upper West regions are likely to accept the capitation system as compared to the Ashanti Region. The findings do not show any significance with regard to the willingness to accept the capitation system for Eastern, Northern, Brong Ahafo and Volta regions.

In addition, the study used the generalized Blinder-Oaxaca decomposition and counterfactual analysis to determine the extent individual characteristics (such as age, gender, educational background, marital status, and high income) influence the willingness and the likelihood of accepting capitation. In this regard, the findings indicate that the willingness and the likelihood to accept the capitation by high-income earners are largely influenced by unexplained factors and not by age, gender, educational background, or marital status.

The study does not find any significance for the willingness to accept the capitation payment system for the Eastern, Northern, Brong Ahafo and Volta regions though the Volta and the Brong Ahafo regions have already been introduced to the pilot program. Also, the success or otherwise of the policy is largely hinged on the income levels of the people irrespective of the individual characteristics. Moreover, any policy that restricts public choice has negative consequences for welfare. The capitation system is a policy that restricts public choice because subscribers are limited to only one service provider. Besides, in the event of subscribers’ emergency ill-health occurring in different vicinity, the subscribers cannot benefit from the health insurance under the capitation system.

Based on the findings and their implications, this study recommends that the capitation system should be rolled out as a complementary payment system to the ongoing payment system of the DRG and not as a replacement. The main purpose of the capitation as a policy measure is to address abuses under the National Health Insurance Scheme, which can be tackled by transforming the current payment system under the DRG and complementing it with the capitation payment system. In the DRG, subscribers have options and varied choices with regard to facilities and service providers. These elements add up to subscribers’ preference for proximity and quality. Furthermore, the abuses which triggered the capitation policy can be addressed by introducing stringent measures such as automation and networking into the current system. This way, the abuses such as multiple claims can be identified and checked.

We recommend that both the DRG and the capitation payment systems be rolled out under the Ghana National Health Insurance Scheme. The capitation payment system should be adopted for general health practitioners providing primary health care while the DRGs should be used to reimburse providers for chronic and acute inpatient care. Thus DRG payment system should be operated under specialist hospitals while capitation payment system be used for primary health care.

In order to check exploitation of the DRG system for supernormal profits, payments should be “case-based” so as to increase efficiency and contain costs. For example, payments for chronic conditions relevant to specific medical condition and treatment should be according to groupings so that an average cost for such chronic conditions be determined in order to check any abuses. As much as possible capitation for inpatients should be separated from outpatients’ fixed annual payments and given different classification for payments and reimbursement. Moreover, incentives should be given to outpatient services to make such services more efficient to avert worsening inpatient situation or transferring patients to specialists’ hospitals that may be costly.

Also, we suggest that prescription by physicians be separated from dispensing by pharmacists/pharmaceutical companies. Similarly, it should be mandatory for copies of prescription and dispensing forms to be submitted to the NHIA by patients for cross-checking and payment with regard to the DRG payment system so as to check the abuses and eliminate any supernormal profits by these parties. When these recommendations are implemented we expect that the two payment systems will be efficient in dealing with excessive cost problems that the NIA (insurer) faces in respect of DRGs and the risk of providing services over and above the per capita payment threshold that service providers receive in the case of capitation. The long hours spent by patients will reduce as patients will now know the type of ailment that should go to the general practitioners and the type that should come to the specialists.

In order to measure properly and deal with changes in the mode of reimbursement, we recommend that the NHIA come up with quality service ratings and quality metrics among peer service providers. The quality measures will inform changes in reimbursement to bring about competition for quality and efficient service delivery. There should be an annual assessment, as well as a review of payments and service delivery. If possible there should be a threshold increase in the yearly maximum reimbursement amount for outpatient visits. This mode of reimbursement can increase outpatient service utilization, which will benefit individuals. Annually, stakeholders must meet to examine and assess all the payment methods with regard to what is working or not and why.

Moreover, the NHIA must examine how it can handle revenue payments over and above revenues generated from premiums as a result of increases and changes in in-out patients. Such attention will inform the NHIA about the consequences and effectiveness of changes in reimbursements. However, where revenues from individual annual-premium-payments, monthly deductions from formal sector workers, and NHIA share from the value added taxation (VAT) are not enough to support the scheme, stakeholders should devise innovative ways of funding. For example, payment of road tolls could be increased to fund health insurance, and the informal sector may be persuaded to contribute to the scheme.

For further research, we propose an extensive qualitative research to elucidate why people prefer certain payment schemes to others. Such qualitative design may apply a flexible in-depth interviews to examine other individual and economic variables which may influence subscribers’ perception and willingness to accept and subscribe to capitation and DRG payment schemes.

## Additional files

Additional file 1: Data. (XLS 279 kb)

Additional file 2: Stata inputs command. (DOCX 11 kb)

## Abbreviations

CHPS: Community-Based Health Planning and Services
DRG: Diagnosis-Related-Grouping
FFS: Fee for Service
GHS: Ghana Health Service
NHIA: National Health Insurance Authority
NHIS: National Health Insurance Scheme
SSNIT: Social Security and National Insurance Trust
