# Supplementary material for: Willingness to accept capitation payment system under the Ghana National Health Insurance Policy: do income levels matter?
Source: Health Econ Rev. 2017 Nov 3;7:38. doi: 10.1186/s13561-017-0175-1 (PMC5670093; doi:10.1186/s13561-017-0175-1)
Supplement: Supplementary file 2 — Stata inputs command. (DOCX 11 kb) [file 13561_2017_175_MOESM2_ESM.docx]

****DETERMINING THE LIKELIHOOD OF ACCEPTING CAPITATION USING VOLTA REGION AS THE BASE CATEGORY

xi:dprobit willing logYlevel i.educatn SEX age awareness employed prference i.maritalstat locatn hholdsize GR ER NR WR BA VR CR UE UW

***DECOMPOSING ACCEPTANCE BASED ON INCOME LEVELS

oaxaca willing hholdsize SEX age employed _Imaritalst_4 _Imaritalst_3 _Imaritalst_2 _Ieducatn_4 _Ieducatn_3 _Ieducatn_2, by( higY) pooled
